# Volatile Compound Profile as a Biomarker of the Ripening Stage of Cornelian Cherry Fruits

**DOI:** 10.3390/molecules31193515

**Published:** 2026-10-02

**Authors:** Agata Antoniewska-Krzeska, Jorge Antonio Custodio-Mendoza, Roksana Szumkowska, Jaroslawa Rutkowska

**Affiliations:** 1 Faculty of Health Sciences, Warsaw University of Life Sciences (WULS-SGGW), Nowoursynowska St. 159c, 02-776 Warsaw, Poland; roksana.szumkowska@gmail.com (R.S.); jaroslawa_rutkowska@sggw.edu.pl (J.R.); 2 Instituto de Agroecoloxía e Alimentación (IAA)—Food and Health Omics, Facultade de Ciencias, Universidade de Vigo—Campus Auga, As Lagoas s/n, 32004 Ourense, Spain; jorgeantonio.custodio@uvigo.gal; 3 Instituto de Materiais (iMATUS), University of Santiago de Compostela, 15782 Santiago de Compostela, Spain

**Keywords:** cornelian cherry (*Cornus mas* L.), fruit maturation, volatile organic compounds, SPME extraction, GC/MS technique

## Abstract

This study aims to evaluate, for the first time, the profile of volatile compounds (VCs) at four different ripening stages of cornelian cherry (*Cornus mas* L. cultivar ‘Bolestraszycki’) fruits: unripe (green), semi-ripe (pale orange and light red), and fully ripe (dark red), to indicate specific volatile markers describing fruit ripening stages. The evolution of aroma VCs was investigated using gas chromatography/mass spectrometry (GC/MS) combined with headspace-SPME extraction. Aldehydes and alcohols dominated the VC profile of cornelian cherry fruits, accounting for 18.2–54.7% and 20.0–45.9%, respectively. The maturation process caused a significant decrease in aldehyde relative amounts (a 7-fold reduction in hexanal) and a simultaneous increase in alcohols (relatively higher amounts of ethanol, butanol, and the presence of hexanol). High levels of hexanal, 2-hexenal (E), pentanol, 1-octen-3-ol, 6-methyl-5-hepten-2-one, camphor, kaurene, cadinene, pentane, and dimethyl sulfide, along with the absence of many esters and alcohols, may serve as volatile markers of unripe fruits. Conversely, high levels of ethanol, butanol, and hexanol, along with numerous esters dominated by ethyl acetate and increased levels of acetic acid, were specific to fully ripe fruits. In conclusion, the presence or relative amount of certain VCs may serve as biomarkers of ripening stages of cornelian cherry fruits.

## 1. Introduction

*Cornus mas* L., also known as the cornelian cherry or dogwood, belongs to the *Cornaceae* family [1]. The cornelian cherry is best known for its edible red-purple fruits with an oval or pear-like shape [2]. The fruits are typically harvested when they have reached full maturity, as indicated by their dark red peel color, soft flesh, and strong aroma. It should be noted that the flavor is very distinctive, characterized as cherry-like and considered either sour or sweet-sour, depending on the cultivar or genotype [2]. Therefore, for nutritional purposes, fully ripe fruits are generally preferred for various product preparations such as jams, juices, compotes, meat sauces, meads, fruit beers, wines, liquors, tinctures (Dereniówka or Drenja with a specific caramel aftertaste), and mixed-fruit preserves. Cornelian cherry fruits can be a valuable functional addition to yogurt, ice cream, white chocolate, and burgers, and their inclusion in products does not decrease consumer acceptability [2,3,4,5,6,7]. Those fruits are rich in organic acids, which contribute to their tartness and make them effective natural acidifiers for various fruit products [8]. The high pectin content in cornelian cherry fruits allows them to be combined with other fruits, such as strawberries, to improve the structure and consistency of jams and marmalades [9]. Compared with other fruits, cornelian cherries are versatile, as they can be used at various stages of ripeness. Interestingly, fermented unripe, fully green fruits are valued as an aperitif and have earned the nickname “Polish olives” [1,2,10] due to their color and flavor. Another use involves red-colored fruits with still firm flesh, harvested just before ripening, which can be used to make sweets [2].

Numerous studies revealed that the cornelian cherry is a rich source of nutritious and biologically active compounds, such as vitamins C, A, E, B_2_, B_7_, polyphenols, and other antioxidants, iridoids, pectin, fiber, organic acids, proteins, lipids, simple sugars (mainly glucose and fructose), and mineral elements (mainly K, Ca, P, Na, Mg, Fe, Zn, Cu, Mn) [3,6,11]. Not only fruits, but also other morphological parts of the cornelian cherry plant provide a wide range of chemical compounds; however, their content and composition differ significantly [11]. Fruit ripening involves a series of complex physiological, biochemical, and structural changes that strongly influence the composition and levels of many attributes that determine the technological suitability of fruits [3,12,13,14,15]. Kucharska and her team’s studies [3,16,17,18] highlight the importance of understanding the chemical composition and content of individual bioactive compounds in cornelian cherry (*Cornus mas* L.) fruits and other plant parts. Recently, the health-promoting attributes, polyphenols, iridoids, and antioxidative properties were also defined during the development and ripening of cornelian cherry (*Cornus mas* L.) fruits at six stages [3].

Fruit aroma is a complex attribute influenced by various pathways, reactions, and numerous volatile organic compounds [14]. The biosynthesis of volatiles in plants is a multi-step, dynamic metabolic process, in which volatile compounds derived from unsaturated fatty acids are produced via the lipoxygenase (LOX) and β-oxidation pathways. In whole fruits, volatiles are generated via β-oxidation, while in disrupted fruit tissue, they form through the LOX pathway. Interestingly, the availability of fatty acids combined with increased membrane permeability, common during fruit ripening, may activate the LOX pathway in intact tissue, potentially serving as an alternative to β-oxidation [14,19]. Additionally, a key step in the biosynthesis of aroma compounds is the presence of primary precursor substrates, such as fatty acids and amino acids, which are highly regulated during fruit development in terms of quantity and composition [20]. C_6_ aldehydes, alcohols, and esters are derived from polyunsaturated fatty acids (PUFAs) and formed via the LOX pathway; for example, C18:2 *9c* and C18:3 *9c12c15c* acids are converted into aroma-active volatiles like short-chain aldehydes, alcohols, and ketones [14]. Recent literature data revealed that fruit maturity significantly influences the composition and abundance of volatile compounds in many fruits, such as peaches, bananas, apples, strawberries, blackberries *Rubus coreanus*, chestnut rose *Rosa roxburghii*, jelly palm *Butia capitata*, mango, and Hongdeng sweet cherry [14,21,22,23,24,25,26,27,28]. It has been indicated that alcohols, aldehydes, and esters are the three major chemical groups of volatiles, common to several fruits. In unripe fruits, C_6_ aldehydes and alcohols are the predominant contributors to ‘green’ fruit aroma but often decrease sharply as the fruit ripens. Meanwhile, esters, lactones, some sulfur volatiles, and sometimes furanones increase in abundance, becoming key contributors to the aroma of mature fruits. These compounds are responsible for a rise in fruity, floral, and sweet aromas [29]. The production of anaerobic metabolites, such as ethanol, in fruits while still on the tree or during postharvest, leads to many changes in fruit ripening, such as the production of aroma volatiles and removal of astringency [30].

To the best of our knowledge, the volatile compound profile of cornelian cherry fruits has not been studied in detail aside from two studies. One of these studies examined the volatile components of mature fruits subjected to hydrodistillation and their pulp (a commercially available product) [31]. In another, volatile compounds were assayed in a homemade alcoholic beverage called “Derenja” made from the ripe fruits [4].

It should also be noted that the study by Spychaj et al. [32], which made the first attempt to valorize cornelian cherry stones through roasting, identified furfural and eighteen pyrazine derivatives as the main aroma compounds. Interestingly, such stones can serve as a substitute for coffee or enhance the aromatic and bioactive richness of cereal coffees. Another study by Yue et al. [33] focused on analyzing the volatiles of *Cornus officinalis* bark powder, which can be utilized more effectively and fully. Among the 276 identified chemical components, ethyne, fluoro- (6.54%), and acetic acid (4.62%) were the most dominant. Yet, these studies do not refer to aromatic changes in fruits during maturation.

However, despite extensive research on the composition and nutritional value of cornelian cherry fruits, no information is available on the volatile compound composition of these fruits. To the best of our knowledge, the evolution of volatile compounds during the ripening of cornelian cherry fruits has not been examined. Therefore, this study aims to characterize the volatile compound profile of cornelian cherry (*Cornus mas* L.) fruits across four ripening stages: unripe (green), semi-ripe (pale orange and light red), and fully ripe (dark red), to identify volatile markers that can be used to track fruit ripening stages. Additionally, to evaluate the stage of ripeness, measurements of titratable acidity, total soluble solids, and in vitro antioxidant capacity of alcoholic extracts of cornelian cherry fruits were also performed.

## 2. Results and Discussion

### 2.1. Physicochemical Characteristics and Antioxidant Properties

Titratable acidity (TA) increased progressively during the maturation of cornelian cherry fruits. Green unripe fruits (RS-1) had the lowest TA, at 6.3 g malic acid (MA)/100 g fruit dry weight (DW), compared with ripe fruits (15.2 and 13.3 g MA/100 g DW). A slight decrease in TA between RS-3 and RS-4 is also worth noting, although it remained considerably higher than in unripe fruits (Table 1). Przybylska et al. [3] also observed an increasing TA pattern with ripening in four *Cornus mas* cultivars (Bolestraszycki, Słowianin, Paczoski, and Florianka), as in kiwifruit. This can be explained by the accumulation of many organic acids, which mostly begins at the early ripening stages and provides substrates used in the respiration of a ripening fruit [3]. Furthermore, organic acids participate in the synthesis of other compounds, including sugars, fatty acids, amino acids, and secondary metabolites. Thus, they are essential for the flavor and quality of fruits [34]. The increase in TA values may be explained by the higher content of malic acid, which dominates the organic acids in ripe cornelian cherry fruits [3]. Similarly, malic acid accounts for about 90% of the total organic acid content of apples [34]. It should be noted that malic acid is the primary compound responsible for the acidity of many fruits, contributing to their tart-sour taste [16]. Additionally, the stability and color of anthocyanins are influenced by the acidity levels. In an acidic environment, anthocyanins exist in the form of the flavylium cation, which is also responsible for the production of the red and purple color of ripe cornelian cherry fruits [35]. The pH values of fruits ranged from 3.13 to 3.56 (Table 1), which is consistent with the acidic character of *Cornus mas* fruits. A decrease in pH was observed from the green (RS-1) to the light-red stage (RS-3), followed by a slight increase at full ripeness (RS-4)—3.35. The pH values obtained in the present study were higher than those reported for cornelian cherry fruits by Demir and Kalyoncu [36] 2.50–2.88, by Gunduz et al. [12] 2.90–3.00, and consistent with those reported by Tural and Koca [37], who found pH values ranging from 3.11 to 3.53.

Total soluble solids (TSSs) varied across the ripening of cornelian cherry fruits, decreasing from 16.8 °Brix in unripe green fruits (RS-1) to 11.5 °Brix at the RS-2 stage, followed by a slight increase to 12.6 °Brix at RS-3. The highest TSS value, 18.4 °Brix, was observed in fully ripe fruits (RS-4). The significant (*p* < 0.05) increase in TSSs observed at the final ripening stage may be explained by the accumulation and concentration of soluble constituents, particularly sugars, which amount increase during fruit maturation steps [3,38]. Our results are in agreement with the study of Przybylska et al. [3] in which TSSs of four different cornelian cherry fruit cultivars fluctuated in all six studied stages, ranging from 14.2–19.0 to 11.0–19.4 °Brix. Also, the study of Gunduz et al. [12] reported an increase in TSS content from light yellow to dark red cornelian cherry fruits.

The antioxidant activity of cornelian cherry fruits was determined using the ABTS^•+^ assay (Table 1). Unripe fruits exhibited a significantly (*p* < 0.05) higher antioxidant activity (98.1 mM TE/100 g DW) than fruits at the later ripening stages, with the activity being 7.3-fold higher than that observed in fully ripe ones (13.5 mM TE/100 g DW). These findings are in agreement with previous reports stating that the antioxidant activity may decrease during fruit ripening, as observed in cornelian cherry fruits, Candonga strawberries, or peach, which is associated with changes in the content and composition of bioactive phenolic compounds during maturation [3,12,15,39,40]. The total phenolic content (TPC) also varied among the ripening stages, maintaining a similar tendency to ABTS^•+^ changes (Table 1). It was found that the highest TPC was in unripe fruits, 1630 mg GAE/100 g FW, and decreased as the cornelian cherry fruit ripened, reaching a level of 367 mg GAE/100 g FW. Celik et al. [41], who investigated the ripening process of cranberry fruits, also reported a decrease in TPC during fruit maturation. Results of our study confirm previous observation about a decrease in phenolic compounds in fruits during maturation stages.

### 2.2. Volatile Compound Profile

The GC/MS analysis revealed a complex mixture of 49 volatile compounds in the headspace of cornelian cherry fruits, including aldehydes (10 compounds), alcohols (10), esters (6), ketones (5), terpenes (11), hydrocarbons (5), and two other compounds (Table 2). Their relative abundance significantly (*p* < 0.05) depended on the degree of maturity. Fully ripe cornelian cherry fruits contained a slightly higher number of volatile compounds (46) than fruits derived from other ripening stages: unripe and intermediate, 39 and 44, respectively.

Regarding relative amounts, aldehydes, which accounted for 18.2 to 49% of the volatile profile, were the most abundant volatile compounds in cornelian cherry fruits. Green, unripe fruit (RS-1) and pale orange intermediately ripe (RS-2) samples were distinguished by about 2-fold higher total relative amount of aldehydes as compared with fruits derived from later stages of ripening (RS-3 and RS-4 samples) (Table 2). It should be noted that many volatiles are produced through processes initiated by cellular disruption, such as cutting or mastication. Among these, six-carbon (C_6_) volatiles—including aldehydes, cis-3-hexenal, its isomer *trans*-2-hexenal, and hexanal—as well as their corresponding alcohols, may be derived from unsaturated fatty acids through the action of lipoxygenase (LOX) and other enzymes in the LOX pathway on substrates released by fruit tissue disruption. During fruit ripening, cell walls and membranes become permeable, allowing LOX to be active without tissue disruption [19,42]. The presence of C_6_ aldehydes in cornelian cherry fruits—hexanal and 2-hexenal (E)—is derived from the process of synthesizing from C18 PUFAs, specifically C18:2 *9c12c* and C18:3 *9c12c15c* fatty acids, via the LOX pathway [43]. Three aldehydes—hexanal, 2-hexenal (E), and nonanal—were identified in analyzed cornelian cherry fruits as the main ones, accounting for 25.2–53.6%, 18.4–19%, and 10.3–14.4% of the total relative abundance of aldehydes during ripening stages, respectively (Table 2). Notably, these C_6_ aldehydes (and alcohols), along with C_9_ aldehydes, contribute to the grassy or green-note aroma and are major contributors to the flavor of immature fruits, according to the literature [14]. Aldehyde levels tend to decrease during fruit maturation, with significant differences [19]. Our study confirmed this trend, consistent with other research on changes during fruit ripening, such as in yellow peach and banana cultivars [14,21]. C_6_ aldehyde compounds are also important contributors to the unripe aroma notes in strawberries and may vary with cultivar and ripeness [44,45]. The low participation of aldehydes in the volatile profile (8.3%) of mature fruits of cornelian cherry (which were subjected to hydrodistillation) was confirmed in the study by Bakirtzi et al. [31].

Alcohols were the second most abundant group of chemicals in the volatile profile of cornelian cherry fruits, with their relative amount ranging from 18% to 43.5%, depending significantly on the maturity stage (*p* < 0.05) (Table 2). This is primarily due to relatively higher levels of ethanol (about 15-fold higher) and butanol (12-fold higher) in fully ripe fruits than in unripe ones. Ethanol is known as a precursor of many aroma compounds. During the maturation and ripening of peaches and nectarines on the tree, an increase in aroma volatiles has been reported, with ethanol and butanol among others as the main components. Also, increased ethanol production in oranges and pears occurred during ripening. However, ethanol is present in relatively low amounts in many apple cultivars; its abundance may increase in over-ripe fruits. Thus, ethanol levels at harvest may serve as a valuable indicator of optimal harvesting time [30]. Matured samples of cornelian cherry fruits (RS-3 and RS-4) were also distinguished from unripe samples (RS-1 and RS-2) by the presence of three new compounds, with hexanol abundance being the predominant one at 9.5%. These results are in accordance with the study by Zhang et al. [28], which investigated the aroma components of Hongdeng sweet cherries and observed that the relative content of hexanol increased during fruit development. Generally, alcohols are reported to contribute to fruity aroma [19]. According to the literature, hexanol is described as having a mild, pleasant, floral-green aroma with fruity and sweet notes [19]. The relative amount of alcohol compounds increased significantly (*p* < 0.05) with fruit ripeness; therefore, their presence in dark red fruits may serve as a good biomarker of the ripe stage of cornelian cherry. Interestingly, unlike other fruits—such as bananas, strawberry trees, or date palms (*Gosbi* cultivar) [21,46,47]—where hexanol decreases during ripening, cornelian cherries show a different pattern. Analyzing the volatile alcohol profile of cornelian cherry fruits, the relative content of some alcohols significantly (*p* < 0.05) decreased during maturation, including pentanol and 1-octen-3-ol, which decreased 6-fold and 4-fold, respectively (Table 2).

According to the literature, esters are associated with pleasant, fruity, and sweet flavors of many ripe fruits [14,19]. In cornelian cherry fruits, six esters were identified (ethyl acetate, butyl acetate, isoamyl acetate, pentyl acetate, butyl butanoate, and hexyl acetate), with their total relative contents significantly (*p* < 0.05) lower (2.7–23.8%) than those of aldehydes and alcohols (Table 2). Ethyl acetate is the dominant ester (2–12.6%) across all samples, regardless of ripening stage. Its presence provides a light, fruity aroma (reminiscent of pears, grapes, and pineapple), sweet, and floral, making it valuable for industrial applications such as flavoring in confections and artificial fruit essences [48]. In mature and ripe guava fruits, levels of ethyl acetate and other esters especially increased [49]. Butyl acetate was identified as the second quantitatively important ester in cornelian cherry fruits, with its relative abundance increasing during maturation (0.7–3.5%). Four other esters were detected only at the later ripening stages (RS-3 and RS-4), and their relative abundance increased as the fruits ripened (Table 2).

Terpenes were represented by twelve compounds. Significant differences (*p* < 0.05) were observed in the relative content of many of them, often depending on the maturity stage (Table 2). The green unripe cornelian cherry fruits (RS-1) were found to be relatively richer in terpene compounds than fully ripe ones, with totals of 12.3% and 2.5%, respectively. Cadinene was detected only in early maturity stages (RS-1 and RS-2), with relative abundances of 4.6% to 5.3%. Cadinene is a bicyclic sesquiterpene (containing 15 carbons derived from three isoprene units) that exhibits a distinct sweet, woody aroma. This volatile compound is widely distributed in plant essential oils, such as grape oil [50], and has antiproliferative and anticancer effects [51]. The other two important terpenes, kaurene and camphor, also showed significant decreases in their relative contents (*p* < 0.05) with fruit maturation (Table 2). The relative abundance of other terpenes does not exceed 1%. However, some terpenes have multiple biological properties. In vitro assays revealed that limonene and α-pinene possess antioxidant activity, may act as potential antidiabetic agents, and have dermatoprotective effects (such as inhibiting tyrosinase and elastase activity) [52].

The five ketones identified in the volatile profile of cornelian cherry fruits showed relatively higher concentrations during early ripening stages compared with aldehydes or alcohols, making them more specific volatiles of ripe fruits (Table 2). The total ketone relative abundance ranged from 3.7% (RS-3) to 6.9% (RS-1), with a dominant share of 6-methyl-5-hepten-2-one. These results align with previous studies on blueberries and pomegranates at different ripening stages, in which ketones were also present at higher levels early on [53,54]. Conversely, Tabaszewska et al. [43] reported a rich abundance of ketones in the volatile profile of *Taxus baccata* L. red arils, which is unusual for many common fruits like apples or pears but may be related to late maturation stages of acerola or mangaba fruits.

The relative amount of hydrocarbons was modest, ranging from 2.9% to 6.3%, and decreased significantly (*p* < 0.05) with maturation. This trend aligns with the volatile profiles of many other fruits [21,23,53]. Pentane was the predominant hydrocarbon, accounting for 79–82% of hydrocarbons. Other hydrocarbons, including styrene, 1-methylethenyl-benzene, undecane, and dodecane, were detected in much lower relative amounts, each not exceeding 0.6% (Table 2). Their presence is unlikely to influence flavor, as hydrocarbons generally have high odor detection thresholds and primarily act as precursors to aromatic compounds [55].

Among other volatiles, acetic acid and dimethyl sulfide were identified. The acetic acid relative amount increased with ripening (0.5–3%), serving as a potential marker of fruit maturity due to the increase in nonvolatile acid levels associated with full ripeness [56]. Likely, acetic acid results from ethanol metabolism. Ethanol can occur as a fermentative by-product, especially when oxygen is limited in fruit tissues. Once present, ethanol can be oxidized to acetic acid through enzymatic and microbial pathways [30]. The presence of acetic acid may contribute to sour, vinegar, and pungent odor notes in many fruits. Significant relative amounts of dimethyl sulfide (2.2–4.4%) were also detected. This compound, along with other sulfur volatiles, has been previously reported in fresh tomatoes and unprocessed fruits like strawberries, durian, and melon. Overall, the contribution of sulfur compounds to aroma depends on their threshold levels and interactions within the food matrix [57].

**Figure 1:**
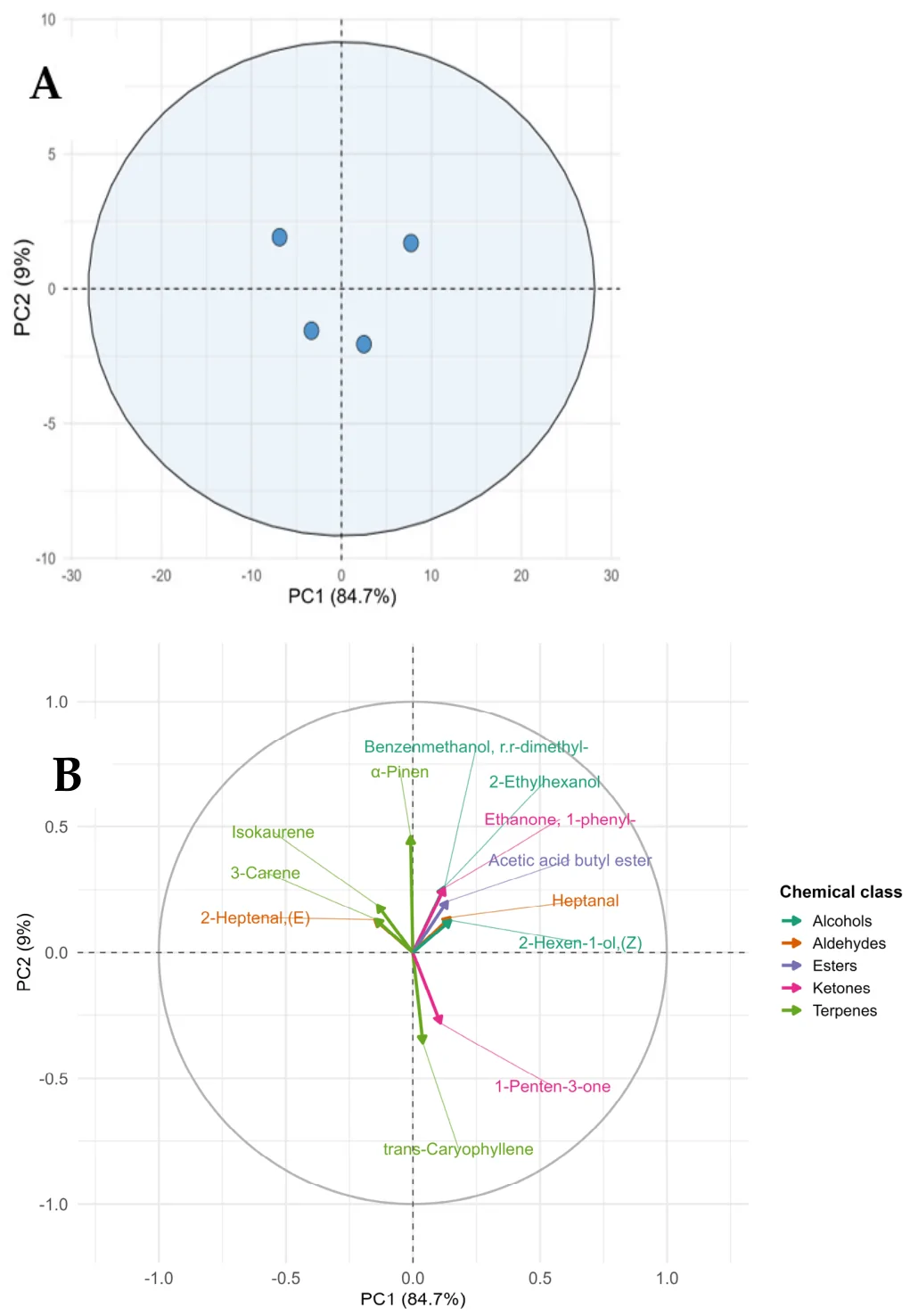
Multivariate analysis of the volatile compound profile of cornelian cherry fruits at different maturity stages. (**A**) Principal component analysis (PCA) score plot showing the distribution of maturity stages. (**B**) PCA loading plot showing the volatile compounds contributing most strongly to PC1 and PC2, colored according to chemical class.

**Figure 2:**
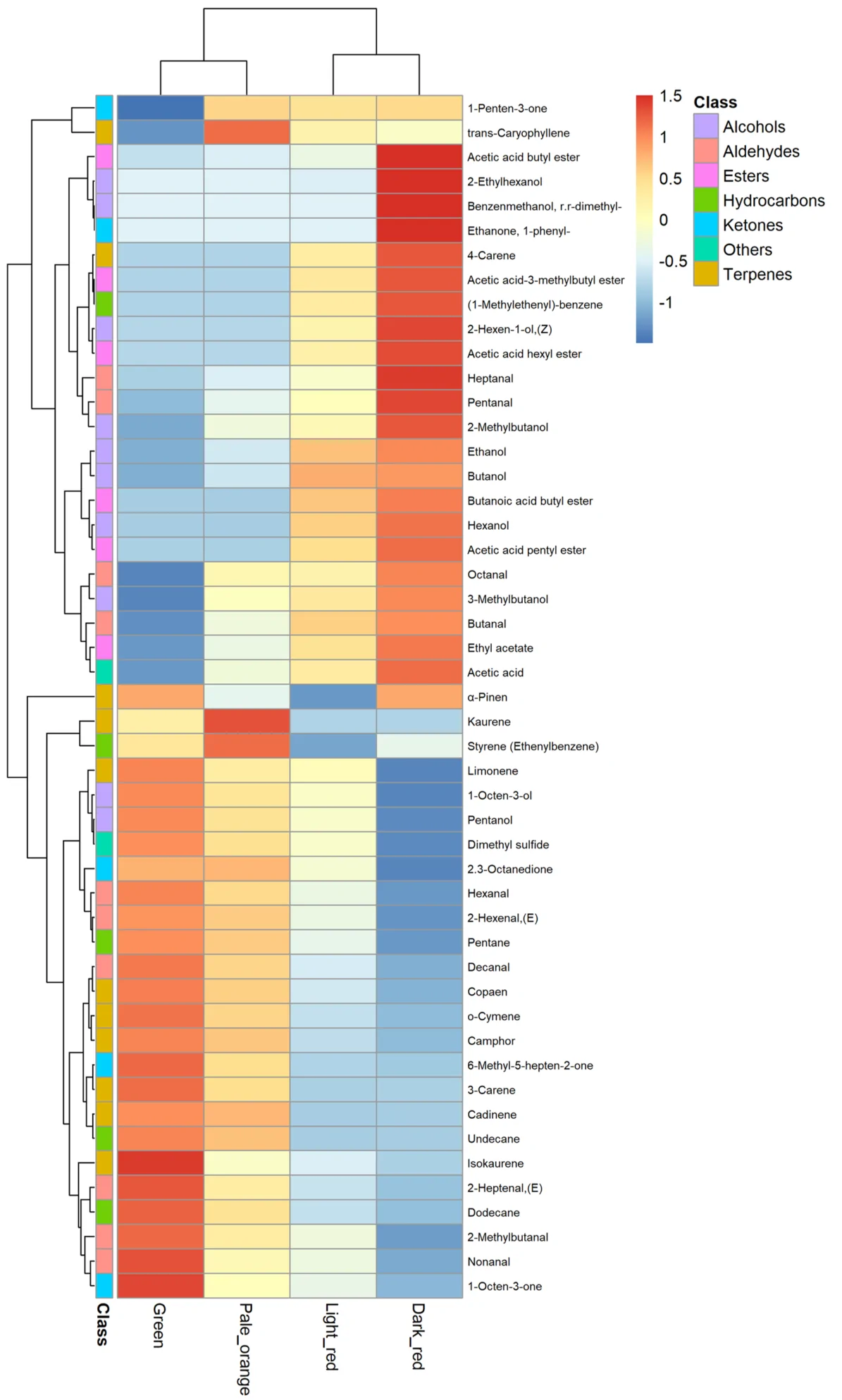
Hierarchically clustered heatmap showing the relative abundance of volatile compounds across different maturity stages of cornelian cherry fruits.

Multivariate statistical analysis revealed pronounced maturity-associated changes in the volatile profile of cornelian cherry fruits (Figure 1 and Figure 2). Principal component analysis (PCA) showed that the first two principal components explained 93.7% of the total variance, with PC1 accounting for 84.7% and PC2 for 9.0% (Figure 1A). The maturity stages occupied distinct positions in the PCA space, with most of the variation occurring along PC1, indicating that fruit ripening was associated with a major coordinated shift in volatile composition. The PCA loading plot showed that this variation resulted from different contributions of individual volatile compounds belonging to different chemical classes (Figure 1B). Several alcohols, aldehydes, esters, and ketones were associated with positive PC1 loadings, whereas isokaurene, 3-carene, and 2-heptenal,(E) were oriented toward negative PC1. Differences along PC2 were particularly influenced by α-pinene, with a strong positive loading, and by *trans*-caryophyllene and 1-penten-3-one, which showed negative PC2 loadings. Overall, the loading distribution indicates that volatile classes did not vary uniformly during ripening but rather followed compound-specific patterns.

The hierarchical clustering heatmap further illustrated the progressive reorganization of the volatile profile during ripening (Figure 2). The green (RS-1) and dark-red (RS-4) stages exhibited markedly contrasting abundance patterns for numerous compounds, whereas the pale-orange (RS-2) and light-red (RS-3) stages generally displayed intermediate profiles. Groups of volatiles showing increased abundance at advanced maturity were accompanied by others showing the opposite trend, demonstrating that ripening involves both accumulation and depletion of specific volatile components. Taken together, these multivariate analyses indicate that maturation of *Cornus mas* L. fruits is accompanied by substantial changes in relative volatile composition. *Trans*-caryophyllene, 2-ethylhexanol, copaene, isokaurene, and 1-penten-3-one are among the volatile features showing pronounced associations with maturity-stage differentiation. However, their potential as specific biomarkers of ripening requires further validation.

**Figure 3:**
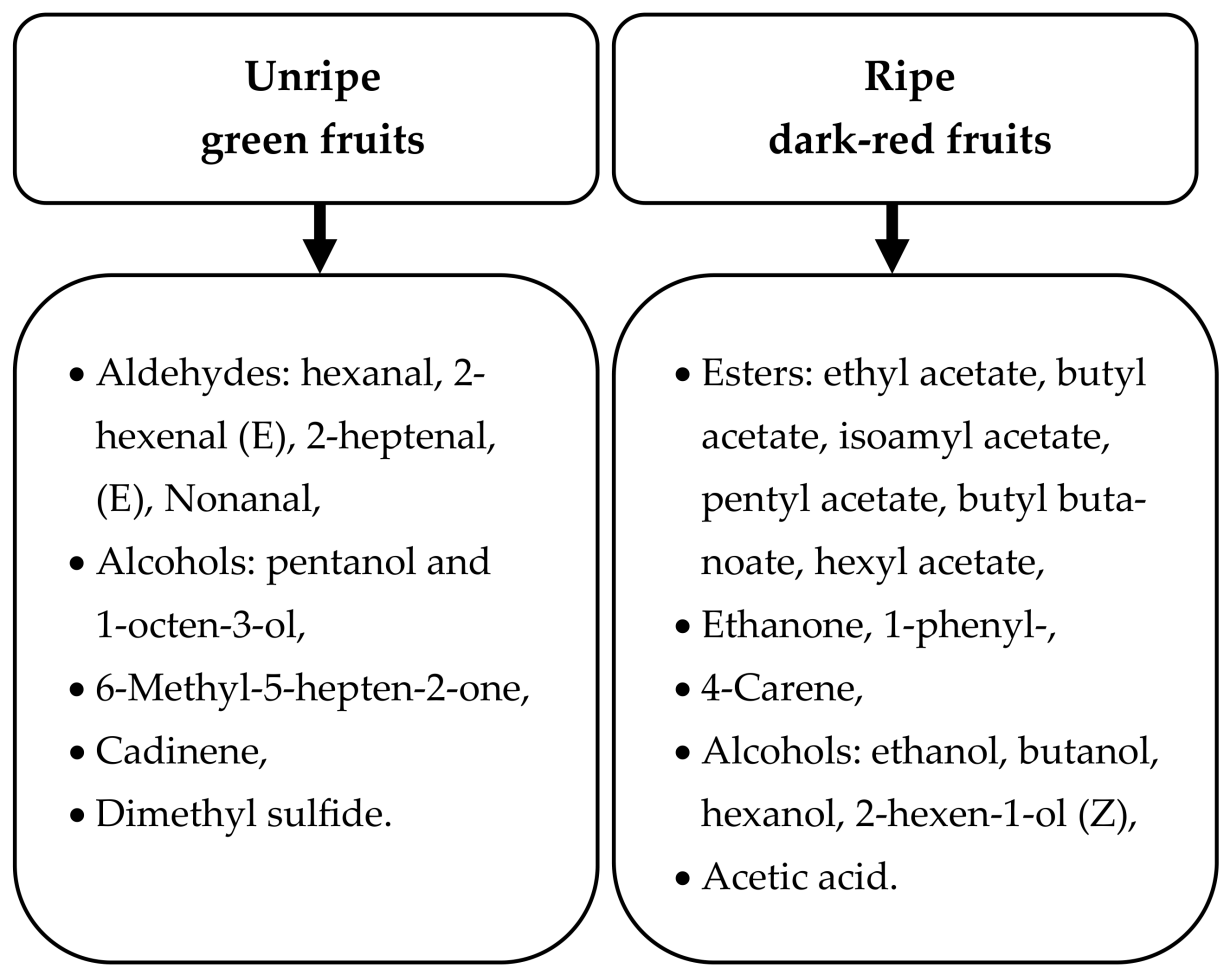
Principal volatile compounds as biomarkers of the ripening stage of cornelian cherry fruits.

In summary, the most significant changes in the volatile profiles of cornelian cherry fruits during ripening were observed in the ester group (Table 2). Maturation was associated with the detection of four new esters not identified at early stages (RS-1 and RS-2): isoamyl acetate, pentyl acetate, butyl butanoate, and hexyl acetate. The maturation process also caused large, statistically significant (*p* < 0.05) changes in the relative contents of aldehydes and alcohols. A substantial decrease in the relative abundance of aldehydes during the ripening process was mainly due to a 6-fold reduction in hexanal relative amounts. At the same time, alcohols increased due to relatively higher levels of ethanol and butanol, as well as the presence of hexanol. Some volatiles (or their relative amounts) were specific to the ripening stage of cornelian cherries, such as relatively high levels of hexanal, 2-hexenal (E), pentanol, 1-octen-3-ol, 6-methyl-5-hepten-2-one, camphor, kaurene, cadinene, pentane, and dimethyl sulfide, along with the absence of many esters and alcohols like hexanol in unripe green fruits (RS-1 samples). Conversely, fully ripe fruits (RS-4) showed relatively high levels of ethanol, butanol, and hexanol, as well as numerous esters (ethyl acetate dominating) and increased acetic acid (Figure 3).

Alongside these findings, a few limitations of the present study should be considered when planning future research. The study provides valuable data on changes in the volatile compound profile during the ripening of cornelian cherry (*Cornus mas* L. cultivar ‘Bolestraszycki’) fruits harvested during a single season and from a single geographical location. Therefore, further research involving other *Cornus mas* cultivars, geographical locations, and multiple growing seasons is needed to better characterize the effects of genetic and seasonal variability on the volatile profile during fruit maturation. Studies integrating these factors will contribute to a more comprehensive understanding of the ripening process and may help determine whether the identified volatiles can serve as robust ripening markers across different cultivars, locations, and seasons.

## 3. Materials and Methods

### 3.1. Fruit Sampling

**Figure 4:**
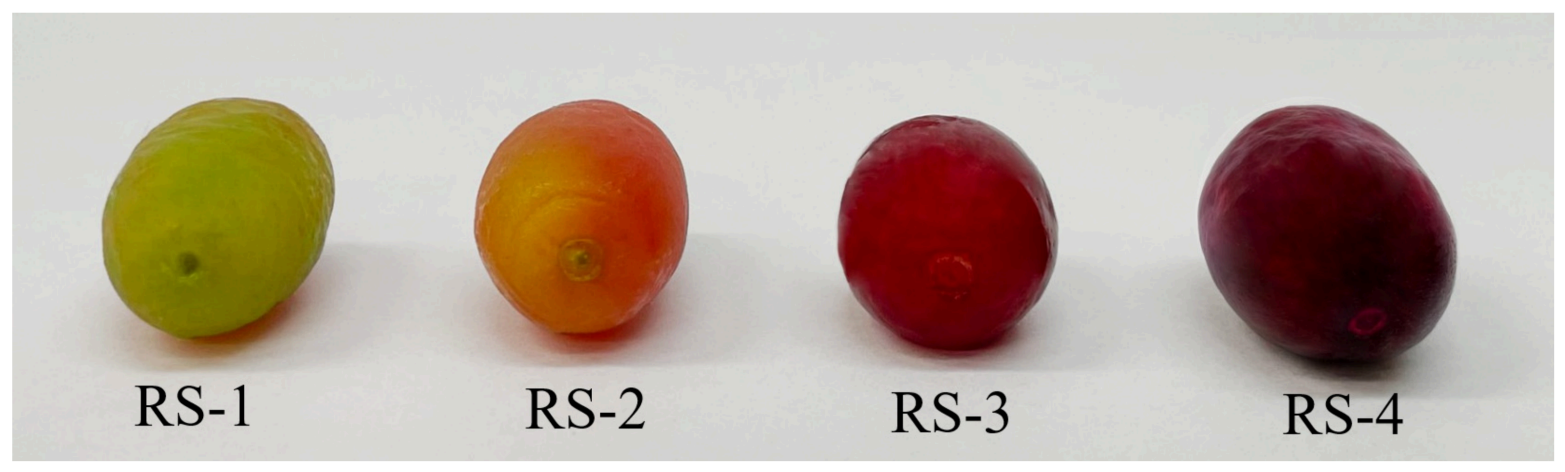
Changes in *Cornus mas* L. fruit color during the studied ripening stages (RS).

The cornelian cherry (*Cornus mas* L. cultivar ‘Bolestraszycki’) fruits were obtained from the Arboretum and Institute of Physiography in Bolestraszyce (near Przemyśl, Poland), located on hilly terrain, at an altitude of 230–250 m above sea level (22°50′ E, 49°54′ N). This area is characterized by a cold temperate continental climate, with average rainfall and annual temperatures of about 663 mm and 7.8 °C, respectively. Total solar radiation is about 1100 kWh/m^2^. Fruits were manually harvested in 2024, at four different ripening stages (Figure 4), namely on 21 July (the unripe fruit with a green peel and firm flesh—ripening stage 1 (RS1), 11 August (the intermediate ripe fruit exhibits a pale orange peel with a red tinge and maintains firm flesh—RS2), 1 September (the fruit in ripening phase exhibits a completely red peel, and the flesh begins to soften—RS3), and 22 September (the peel turns dark red, the texture is very soft, appeared intensified aroma, and the fruits were categorized as fully ripe—RS4). The plant materials were authenticated by experienced botanist Ewa Antoniewska, M.Sc. A total of 100 fruits at each ripening stage were harvested (from different parts of the plant, from five different plants). The fruits exhibited no visual symptoms of any disease or blemishes. Collected fruits were transferred to the laboratory, surface-cleaned with a clean and dry cotton cloth, washed in tap water, and air-dried at ambient temperature (18 °C). The skin and flesh were separated from the stones, homogenized (Zelmer, Rzeszów, Poland), and analyzed.

### 3.2. Titratable Acidity and pH Value

The titratable acidity (TA) was determined according to Przybylska et al. [3] by titrating homogenized fruit pulp with 0.1 N NaOH to a slightly alkaline endpoint (pH 8.1) using a CP-411 pH meter (Elmetron Company, Zabrze, Poland) with a combined electrode. The results were expressed as g of malic acid per 100 g of the fruit sample.

The pH value of the homogenized fruit pulp was determined potentiometrically using a pH meter equipped with a combined electrode at ambient temperature.

### 3.3. Total Soluble Solids Content

Fresh fruits were deseeded, then homogenized, and the juice was obtained by gentle pressing through gauze. The total soluble solid (TSS) content was measured using an Abbe Refractometer AR4 (A. KRÜSS Optronic GmbH, Hamburg, Germany) and expressed as °Brix.

### 3.4. Antioxidant Activity—ABTS^•+^ Assay and Total Phenolics Content

For the spectrophotometric assays, cornelian cherry fruit extracts were prepared by mixing 10 g of fruit samples with 100 mL of an ethanol-water mixture (80:20, *v*/*v*), followed by homogenization using a DI 25 homogenizer (Ika-Werke, Staufen, Germany). The samples were subsequently centrifuged at 15,000 rpm for 20 min using an MPW-260R centrifuge (MPW Med. Instruments, Warsaw, Poland), and the resulting supernatants were collected and used further.

Antioxidant activity of the binary hydroalcoholic extracts of cornelian cherry fruits was measured spectrophotometrically at 734 nm (UV-Vis spectrophotometer Specord 40, Analytik Jena AG, Jena, Germany) using a radical cation scavenging assay ABTS^•+^, according to Re et al. [58] and Tabaszewska et al. [43]. The results were expressed as Trolox equivalents (TE) in mM/100 g of the fruit’s dry weight.

The total phenolic content (TPC) was determined spectrophotometrically at 765 nm (UV-Vis spectrophotometer Specord 40, Analytik Jena AG, Jena, Germany) using the reduction in Folin–Ciocalteu’s reagent to blue pigments in alkaline solution according to a procedure presented previously [43]. The results were expressed as gallic acid equivalents (GAE; mg GAE/100 g FW).

### 3.5. Analysis of Volatile Compound Profile

Volatile compounds were extracted from fruit pulp samples collected at four different ripening stages using a previously reported procedure with minor modifications [43]. Briefly, 5 g of each fruit sample was placed in a 20 mL glass vial, closed with a silicone-Teflon sealing cap, and volatile compounds were extracted by headspace solid-phase microextraction (HS-SPME) using a triple-coated fiber (50/30 µm DVB/CAR/PDMS, Supelco, Bellefonte, PA, USA). The SPME fiber was inserted into the vial headspace and exposed to the sample for 40 min at 40 °C. Following extraction, the fiber was thermally desorbed in the gas chromatograph (GC) injection port at 270 °C for 5 min.

Gas chromatographic separation was performed using an Agilent 6890N GC coupled to a 5975 mass selective (MS) detector (Agilent Technologies, Santa Clara, CA, USA). Volatile compounds were separated on an HP-5MS capillary column (30 m × 0.25 mm i.d., 0.25 μm film thickness; 5%-diphenyl/95%-polydimethylsiloxane; Agilent Technologies) under splitless injection conditions. Helium (99.999% purity) was used as the carrier gas at a constant flow rate of 0.9 mL/min. The oven temperature was initially set at 38 °C and maintained for 10 min, followed by an increase to 200 °C at a rate of 4 °C/min and a 2 min hold. The temperature was subsequently increased to 250 °C at 20 °C/min and maintained for 7 min. The total chromatographic run time was 62 min.

The MS was operated in electron ionization (EI) mode at an ionization energy of 70 eV. Data were acquired in full-scan mode over an *m*/*z* range of 30–350, with the ion source temperature maintained at 230 °C. Volatile compounds were identified based on their mass spectral fragmentation patterns, relative ion intensities, retention times (RTs), and linear retention indices (LRIs). Mass spectra were compared with those contained in the NIST 08 Mass Spectral Search Program and Wiley 8th Edition spectral libraries. LRIs were calculated according to the method of Van den Dool and Kratz [59] using a homologous series of n-alkanes (C_6_-C_20_; Sigma-Aldrich, Poznań, Poland) analyzed under identical chromatographic conditions. The relative abundance of individual volatile compounds was expressed as a percentage of the total peak area, calculated as follows: relative abundance (%) = (peak area of an individual volatile compound/total peak area of all identified compounds) × 100. Each sample assay consisted of three replicates.

### 3.6. Statistical Analysis

The results were presented as means ± standard deviations (SD) and analyzed using a one-factor analysis of variance (ANOVA), followed by Tukey’s test. Differences between mean values were considered significant at *p* < 0.05. Multivariate analysis of the volatile-compound dataset included principal component analysis (PCA) and hierarchical cluster analysis (HCA). PCA was used as an unsupervised exploratory approach to evaluate the overall variation in volatile profiles among maturity stages and to identify the compounds contributing most strongly to this variation. Hierarchical clustering combined with heatmap visualization was used to assess similarities among maturity stages and patterns of relative volatile abundance. Prior to multivariate analysis, volatile-compound data were standardized to account for differences in scale among variables; for heatmap visualization, individual compounds were additionally mean-centered and scaled to unit variance (z-scores) across maturity stages. Hierarchical clustering was performed using Euclidean distance and complete linkage. The Statistica 3.1 software (Statsoft, Inc., Tulsa, OK, USA) was used. All analyses were performed in triplicate, while multivariate analyses were performed using R [R Core Team, version 4.5.0], using the packages factoextra, pheatmap, ggplot2, and dplyr.

## 4. Conclusions

To the authors’ knowledge, this is the first documented volatile profile of cornelian cherry (*Cornus mas* L.) fruits during maturation. Our study demonstrates that the volatile composition of cornelian cherry fruits is significantly affected by ripening; therefore, volatiles can potentially serve as real-time biomarkers for identifying the ripening stage. In particular, increased relative levels of ethanol, butanol, and hexanol, together with the occurrence of several esters, primarily ethyl acetate, and relatively higher levels of acetic acid, characterized the more advanced maturity stages. Multivariate analysis further highlighted compounds such as *trans*-caryophyllene, 2-ethylhexanol, copaene, isokaurene, and 1-penten-3-one as potential contributors associated with maturity stages. These findings indicate that SPME-GC/MS profiling can provide a useful analytical approach for characterizing fruit maturity and identifying volatile features associated with ripening. With further validation, such volatile signatures could support maturity assessment and harvest-time optimization, as well as quality evaluation of cornelian cherry fruits intended for processing and for fruit sellers.

## Figures and Tables

**Table 1:** Changes in the physicochemical attributes during ripening of cornelian cherry fruits (means ± SD).

	Fruit Ripening Stages			
	Unripe	Intermediate Ripe		Fully Ripe
	Green (RS-1)	Pale Orange (RS-2)	Light Red (RS-3)	Dark Red (RS-4)
TA, g MA/100 g DW	6.25 ± 0.08 ^a^	9.54 ± 0.13 ^b^	15.21 ± 0.28 ^d^	13.33 ± 0.21 ^c^
pH	3.56 ± 0.05 ^c^	3.13 ± 0.03 ^a^	3.20 ± 0.03 ^a^	3.35 ± 0.02 ^b^
TSS, °Brix	16.8 ± 0.3 ^b^	11.5 ± 0.2 ^a^	12.6 ± 0.5 ^a^	18.4 ± 0.4 ^c^
ABTS^•+^, mM TE/100 g DW	98.05 ± 3.18 ^d^	66.21 ± 1.42 ^c^	22.84 ± 0.65 ^b^	13.46 ± 0.31 ^a^
TPC, mg GAE/100 g FW	1630 ± 22 ^d^	1163 ± 37 ^c^	821 ± 29 ^b^	367 ± 19 ^a^

^a,b,c,d^—The same letters in the row indicate the lack of a significant difference at *p* < 0.05. TA—titratable acidity; TSS—Total soluble solid; TPC—Total phenolic content; GAE—Gallic acid equivalent; TE—Trolox equivalent; DW—Dry weight; FW—Fresh weight; RS—Ripening stage.

**Table 2:** Relative abundance of volatile compounds (%) in cornelian cherry fruits at different ripening stages (means ± SD).

	RT	LRI	Compound	Relative Volatile Compound Abundance (%) in Fruits			
				Unripe	Intermediate Ripe	Fully Ripe	
				Green (RS-1)	Pale Orange (RS-2)	Light Red (RS-3)	Dark Red (RS-4)
Aldehydes							
1	2.040	592.7	Butanal	1.07 ± 0.08 ^a^	1.66 ± 0.05 ^a^	2.12 ± 0.03 ^b^	2.34 ± 0.08 ^c^
2	2.063	595.9	2-Methylbutanal	2.27 ± 0.06 ^d^	1.65 ± 0.03 ^c^	1.27 ± 0.02 ^b^	0.62 ± 0.01 ^a^
3	3.405	691.2	Pentanal	0.56 ± 0.05 ^a^	0.67 ± 0.03 ^b^	0.75 ± 0.02 ^b^	1.00 ± 0.02 ^c^
4	6.546	796.3	Hexanal	26.23 ± 2.93 ^d^	21.44 ± 1.55 ^c^	13.57 ± 0.65 ^b^	4.58 ± 0.38 ^a^
5	9.829	847.5	2-Hexenal, (E)	9.31 ± 0.71 ^c^	8.45 ± 0.54 ^c^	5.91 ± 0.20 ^b^	3.34 ± 0.38 ^a^
6	13.343	900.1	Heptanal	0.44 ± 0.06 ^a^	0.49 ± 0.03 ^a^	0.56 ± 0.07 ^a^	0.81 ± 0.14 ^b^
7	16.623	957.4	2-Heptenal, (E)	2.52 ± 0.15 ^c^	1.62 ± 0.11 ^b^	0.80 ± 0.05 ^a^	0.53 ± 0.02 ^a^
8	19.145	1001.4	Octanal	0.94 ± 0.11 ^a^	1.49 ± 0.05 ^b^	1.52 ± 0.02 ^b^	1.83 ± 0.04 ^c^
9	23.793	1103.1	Nonanal	5.02 ± 0.28 ^c^	3.84 ± 0.23 ^b^	3.44 ± 0.10 ^b^	2.62 ± 0.08 ^a^
10	27.823	1204.8	Decanal	0.60 ± 0.03 ^b^	0.58 ± 0.02 ^b^	0.54 ± 0.00 ^a^	0.52 ± 0.01 ^a^
Total aldehydes				48.96 ± 1.63 ^d^	41.89 ± 0.83 ^c^	30.48 ± 0.69 ^b^	18.19 ± 0.51 ^a^
Alcohols							
1	1.660	549.2	Ethanol	0.74 ± 0.02 ^a^	3.24 ± 0.35 ^b^	9.43 ± 0.82 ^c^	11.14 ± 1.81 ^d^
2	2.904	650.6	Butanol	0.77 ± 0.05 ^a^	2.83 ± 0.12 ^b^	8.71 ± 0.35 ^c^	9.22 ± 0.74 ^c^
3	4.269	724	3-Methylbutanol	0.66 ± 0.01 ^a^	1.35 ± 0.05 ^b^	1.54 ± 0.08 ^b^	1.88 ± 0.08 ^c^
4	4.378	727.3	2-Methylbutanol	0.47 ± 0.06 ^a^	1.46 ± 0.05 ^b^	1.89 ± 0.10 ^c^	3.22 ± 0.31 ^d^
5	5.275	756.0	Pentanol	6.41 ± 0.43 ^d^	5.16 ± 0.15 ^c^	3.88 ± 0.06 ^b^	1.14 ± 0.02 ^a^
6	11.085	866.4	2-Hexen-1-ol, (Z)	nd	nd	0.40 ± 0.03 ^a^	0.92 ± 0.06 ^b^
7	11.262	870	Hexanol	nd	nd	6.87 ± 0.11 ^a^	9.47 ± 1.83 ^b^
8	18.014	981.5	1-Octen-3-ol	8.66 ± 0.30 ^d^	6.98 ± 0.21 ^c^	5.67 ± 0.08 ^b^	2.05 ± 0.10 ^a^
9	20.494	1030.9	2-Ethylhexanol	0.30 ± 0.00 ^b^	0.32 ± 0.02 ^b^	0.24 ± 0.01 ^a^	3.69 ± 0.08 ^c^
10	22.922	1083.8	Benzenmethanol, dimethyl-	nd	nd	nd	0.78 ± 0.02
Total alcohols				18.01 ± 0.16 ^a^	21.34 ± 0.20 ^a^	38.63 ± 0.31 ^b^	43.51 ± 1.15 ^c^
Esters							
1	2.376	607.7	Ethyl acetate	2.04 ± 0.05 ^a^	6.24 ± 0.08 ^b^	9.68 ± 0.16 ^c^	12.58 ± 0.42 ^d^
2	7.447	801.3	Butyl acetate	0.65 ± 0.02 ^a^	0.85 ± 0.05 ^b^	1.11 ± 0.02 ^c^	3.52 ± 0.10 ^d^
3	11.831	877.5	Isoamyl acetate	nd	nd	1.38 ± 0.00 ^a^	2.49 ± 0.02 ^b^
4	14.387	918.3	Pentyl acetate	nd	nd	0.24 ± 0.01 ^a^	0.36 ± 0.02 ^b^
5	18.915	997.1	Butyl butanoate	nd	nd	0.76 ± 0.03 ^a^	0.96 ± 0.05 ^b^
6	19.797	1015.6	Hexyl acetate	nd	nd	1.86 ± 0.00 ^a^	3.88 ± 0.02 ^b^
Total esters				2.69 ± 0.03 ^a^	7.09 ± 0.06 ^b^	15.03 ± 0.04 ^c^	23.79 ± 0.13 ^d^
Ketones							
1	3.239	683	1-Penten-3-one	1.07 ± 0.05 ^a^	1.70 ± 0.03 ^b^	1.66 ± 0.02 ^b^	1.69 ± 0.00 ^b^
2	17.893	979.3	1-Octen-3-one	1.07 ± 0.02 ^d^	0.54 ± 0.02 ^c^	0.39 ± 0.00 ^b^	0.12 ± 0.00 ^a^
3	18.297	986.4	2.3-Octanedione	1.10 ± 0.01 ^c^	1.09 ± 0.03 ^c^	0.68 ± 0.02 ^b^	0.14 ± 0.01 ^a^
4	18.387	987.7	6-Methyl-5-hepten-2-one	3.61 ± 0.05 ^c^	2.68 ± 0.00 ^b^	0.92 ± 0.01 ^a^	0.81 ± 0.03 ^a^
5	22.029	1064.3	Ethanone, 1-phenyl-	nd	nd	nd	1.22 ± 0.02
Total ketones				6.85 ± 0.03 ^c^	6.01 ± 0.02 ^b^	3.65 ± 0.01 ^a^	3.98 ± 0.01 ^a^
Terpenes							
1	17.471	1027.2	α-Pinene	0.65 ± 0.03 ^d^	0.55 ± 0.03 ^c^	0.48 ± 0.02 ^b^	0.65 ± 0.00 ^a^
2	19.299	972.0	3-Carene	0.15 ± 0.00 ^a^	0.14 ± 0.00 ^a^	0.12 ± 0.01 ^a^	0.12 ± 0.01 ^a^
3	20.061	1004.9	o-Cymene	0.31 ± 0.03 ^b^	0.26 ± 0.01 ^b^	0.15 ± 0.02 ^a^	0.12 ± 0.00 ^a^
4	20.242	1021.6	Limonene	0.84 ± 0.90 ^b^	0.74 ± 0.03 ^b^	0.71 ± 0.05 ^b^	0.52 ± 0.01 ^a^
5	25.305	1025.4	Camphor	1.91 ± 0.16 ^c^	2.33 ± 0.11 ^d^	0.44 ± 0.05 ^b^	0.12 ± 0.01 ^a^
6	30.745	1141	4-Carene	nd	nd	0.11 ± 0.01 ^a^	0.21 ± 0.00 ^b^
7	33.765	1306.4	Copaen	0.25 ± 0.00 ^b^	0.23 ± 0.02 ^b^	0.18 ± 0.00 ^a^	0.16 ± 0.00 ^a^
8	35.182	1380.3	Caryophyllene	0.20 ± 0.05 ^a^	0.30 ± 0.00 ^b^	0.26 ± 0.02 ^b^	0.25 ± 0.03 ^b^
9	50.831	1419.3	Isokaurene	0.60 ± 0.06 ^b^	0.46 ± 0.02 ^a^	0.42 ± 0.00 ^a^	0.39 ± 0.01 ^a^
10	52.027	1991.8	Kaurene	1.69 ± 0.07 ^a^	3.40 ± 0.14 ^b^	nd	nd
11	38.402	2041.0	Cadinene	5.28 ± 0.62 ^a^	4.55 ± 1.03 ^b^	nd	nd
Total terpenes				12.30 ± 0.23 ^b^	12.54 ± 0.18 ^c^	2.87 ± 0.05 ^a^	2.54 ± 0.02 ^a^
Hydrocarbons							
1	1.758	557.5	Pentane	5.22 ± 0.61 ^c^	4.75 ± 0.15 ^c^	3.42 ± 0.03 ^b^	2.27 ± 0.01 ^a^
2	12.355	885.4	Styrene (Ethenylbenzene)	0.26 ± 0.00 ^a^	0.27 ± 0.02 ^a^	0.24 ± 0.03 ^a^	0.25 ± 0.01 ^a^
3	17.931	979.9	(1-Methylethenyl)-benzene	nd	nd	0.12 ± 0.00 ^a^	0.22 ± 0.03 ^b^
4	23.612	1098.8	Undecane	0.60 ± 0.05 ^b^	0.49 ± 0.01 ^a^	nd	nd
5	27.605	1198.7	Dodecane	0.26 ± 0.03 ^b^	0.21 ± 0.00 ^b^	0.14 ± 0.01 ^a^	0.12 ± 0.03 ^a^
Total hydrocarbons				6.34 ± 0.26 ^c^	5.72 ± 0.31 ^c^	3.92 ± 0.03 ^b^	2.86 ± 0.09 ^a^
Others							
1	2.327	603.9	Acetic acid	0.46 ± 0.02 ^a^	1.51 ± 0.01 ^b^	2.07 ± 0.03 ^b^	2.95 ± 0.06 ^c^
2	1.841	564.2	Dimethyl sulfide	4.39 ± 0.11 ^c^	3.90 ± 0.15 ^c^	3.35 ± 0.08 ^b^	2.18 ± 0.02 ^a^

^a,b,c,d^—The same letters in the row indicate the lack of a significant difference at *p* < 0.05. RT—retention time (min); LRI—linear retention index value; nd—not detectable; RS—Ripening stage.

## Data Availability

The original contributions presented in this study are included in the article. Further inquiries can be directed to the corresponding author.

## References

[1] Szot I., Szot P., Lipa T., Sosnowska B., Dobrzański B. (2019). Determination of physical and chemical properties of cornelian cherry (*Cornus mas* L.) fruits depending on degree of ripening and ecotypes. Acta Sci. Pol. Hortorum Cultus.

[2] Szczepaniak O.M., Kobus-Cisowska J., Kusek W., Przeor W. (2019). Functional properties of cornelian cherry (*Cornus mas* L.): A comprehensive review. Eur. Food Res. Technol..

[3] Przybylska D., Kucharska A.Z., Piórecki N., Sozański T. (2024). The health-promoting quality attributes, polyphenols, iridoids and antioxidant activity during the development and ripening of cornelian cherry (*Cornus mas* L.). Antioxidants.

[4] Tešević V., Nikićević N., Milosavljević S., Bajić D., Vajs C., Vučković I., Vujisić L., Dordević I., Stanković M., Veličković M. (2009). Characterization of volatile compounds of “Drenja”, an alcoholic beverage obtained from the fruits of cornelian cherry. J. Serb. Chem. Soc..

[5] Jurečková Z., Diviš P., Cetkovská J., Vespalcová M., Pořízka J., Řezníček V. (2021). Fruit characteristics of different varieties of cornelian cherry (*Cornus mas* L.) cultivated in the Czech Republic. Erwerbs-Obstbau.

[6] Adamenko K., Kawa-Rygielska J., Kucharska A.Z., Piórecki N. (2018). Characteristics of biologically active compounds in cornelian cherry meads. Molecules.

[7] Tarko T., Duda-Chodak A., Satora P., Sroka P., Pogoń P., Machalica J. (2014). *Chaenomeles japonica*, *Cornus mas*, *Morus nigra* fruits characteristics and their processing potential. J. Food Sci. Technol..

[8] Nawirska-Olszańska A., Biesiada A., Sokół-Łętowska A., Kucharska A.Z. (2011). Content of bioactive compounds and antioxidant capacity of pumpkin puree enriched with Japanese quince, cornelian cherry, strawberry and apples. Acta Sci. Pol. Technol. Aliment..

[9] Rop O., Mlcek J., Kramarova D., Jurikova T. (2010). Selected cultivars of cornelian cherry (*Cornus mas* L.) as a new food source for human nutrition. Afr. J. Biotechnol..

[10] Kucharska A.Z., Piórecki N., Sokół-Łętowska A., Żarowska B. (2011). Characteristics of chemical composition and antioxidant properties of cornelian cherry fruit fermented in brine. Zesz. Probl. Postępów Nauk Rol..

[11] Antoniewska-Krzeska A., Ivanišová E., Klymenko S., Bieniek A.A., Fatrcová-Šramková K., Brindza J. (2022). Nutrients content and composition in different morphological parts of cornelian cherry (*Cornus mas* L.). Agrobiodiversity Improv. Nutr. Health Life Qual..

[12] Gunduz K., Saracoglu O., Özgen M., Serce S. (2013). Antioxidant, physical and chemical characteristics of cornelian cherry fruits (*Cornus mas* L.) at different stages of ripeness. Acta Sci. Pol. Hortorum Cultus.

[13] Castro R.I., Vásquez-Rojas C., Cortiella M.G.I., Parra-Palma C., Ramos P., Morales-Quintana L. (2023). Evolution of the volatile organic compounds, phenols and antioxidant capacity during fruit ripening and development of *Rubus ulmifolius* Schott fruits. Horticulturae.

[14] Liu H., Zhang M., Su M., Zeng W., Wang S., Du J., Zhou H., Yang X., Zhang X., Li X. (2025). Multidimensional analysis of the flavor characteristics of yellow peach at different ripening stages: Chemical composition profiling and sensory evaluation. Food Chem..

[15] Cozzolino R., Pace B., Palumbo M., Laurino C., Picariello G., Siano F., De Giulio B., Pelosi S., Cefola M. (2021). Profiles of volatile and phenolic compounds as markers of ripening stage in Candonga strawberries. Foods.

[16] Kucharska A.Z. (2012). Active Compounds of Cornelian Cherry Fruit (Cornus mas L.).

[17] Kucharska A.Z., Szumny A., Sokół-Łętowska A., Piórecki N., Klymenko S.V. (2015). Iridoids and anthocyanins in cornelian cherry (*Cornus mas* L.) cultivars. J. Food Compos. Anal..

[18] Klymenko S., Kucharska A.Z., Sokół-Łętowska A., Piórecki N., Przybylska D., Grygorieva O. (2021). Iridoids, flavonoids, and antioxidant capacity of *Cornus mas*, *C. officinalis*, and *C. mas* × *C. officinalis* fruits. Biomolecules.

[19] El Hadi M.A.M., Zhang F.-J., Wu F.-F., Zhou C.-H., Tao J. (2013). Advances in Fruit Aroma Volatile Research. Molecules.

[20] Song J., Bangerth F. (2003). Fatty acids as precursors for aroma volatile biosynthesis in pre-climacteric and climacteric apple fruit. Postharvest Biol. Technol..

[21] Zhu X., Li Q., Li J., Luo J., Chen W., Li X. (2018). Comparative study of volatile compounds in the fruit of two banana cultivars at different ripening stages. Molecules.

[22] Ma J., Li X., Chu Y., Yue H., Xu Z., Li B., Wu X., Gan J., Jia Y. (2024). Characterization of changes in ripening process of volatile apple compounds based on HS-SPME-GC-MS analysis. Agriculture.

[23] Padilla-Jimenez S.M., Angoa-Pérez M.V., Mena-Violante H.G., Oyoque-Salcedo G., Montañez-Soto J.L., Oregel-Zamudio E. (2021). Identification of organic volatile markers associated with aroma during maturation of strawberry fruits. Molecules.

[24] Yu A.-N., Yang Y.-N., Yang Y., Zheng F.-P., Sun B.-G. (2019). Free and bound volatile compounds in the *Rubus coreanus* fruits of different ripening stages. J. Food Biochem..

[25] Xu S., Deng J., Wu S., Fei Q., Lin D., Chen H., Tao G., Meng L., Hu Y., Ma F. (2024). Dynamic changes of active components and volatile organic compounds in *Rosa roxburghii* fruit during the process of maturity. Foods.

[26] Aguiar M.C.S., Silvério F.O., de Pinho G.P., Lopes P.S.N., Fidêncio P.H., Ventura S.J. (2014). Volatile compounds from fruits of *Butia capitata* at different stages of maturity and storage. Food Res. Int..

[27] Pandit S.S., Kulkarni R.S., Chidley H.G., Giri A.P., Pujari K.H., Köllner T.G., Degenhardt J., Gershenzon J., Gupta V.S. (2009). Changes in volatile composition during fruit development and ripening of ‘Alphonso’ mango. J. Sci. Food Agric..

[28] Zhang X., Jiang Y.-M., Peng F.-T., He N.-B., Li Y.-J., Zhao D.-C. (2007). Changes of aroma components in Hongdeng sweet cherry during fruit development. Agric. Sci. China.

[29] Defilippi B.G., Manríquez D., Luengwilai K., González-Agüero M. (2009). Chapter 1 Aroma volatiles: Biosynthesis and mechanisms of modulation during fruit ripening. Adv. Bot. Res..

[30] Pesis E. (2005). The role of the anaerobic metabolites, acetaldehyde and ethanol, in fruit ripening, enhancement of fruit quality and fruit deterioration. Postharvest Biol. Technol..

[31] Bakirtzi C., Tsatalas P., Spanakis M., Kokkalou E. (2013). GC-MS Analysis of volatile constituents of *Cornus mas* fruits and pulp. J. Essent. Oil-Bear. Plants.

[32] Spychaj R., Kucharska A.Z., Szumny A., Przybylska D., Pejcz E., Piórecki N. (2021). Potential valorization of Cornelian cherry (*Cornus mas* L.) stones: Roasting and extraction of bioactive and volatile compounds. Food Chem..

[33] Yue X., Li X., Chen X., Ashraf M.A., Liu Z., Bi H., Zheng D., Zhao Y., Peng W. (2018). Molecules and functions of *Cornus officinalis* bark volatiles. Emir. J. Food Agric..

[34] Zheng L., Ma W., Liu P., Song S., Wang L., Yang W., Ren H., Wei X., Zhu L., Peng J. (2024). Transcriptional factor MdESE3 controls fruit acidity by activating genes regulating malic acid content in apple. Plant Physiol..

[35] Enaru B., Drețcanu G., Pop T.D., Stǎnilǎ A., Diaconeasa Z. (2021). Anthocyanins: Factors affecting their stability and degradation. Antioxidants.

[36] Demir F., Kalyoncu I.H. (2003). Some Nutritional, Pomological and Physical Properties of Cornelian Cherry (*Cornus mas* L.). J. Food Eng..

[37] Tural S., Koca I. (2008). Physico-chemical and antioxidant properties of cornelian cherry fruits (*Cornus mas* L.) grown in Turkey. Sci. Hortic..

[38] Phan A.D.T., Zhang J., Seididamyeh M., Srivarathan S., Netzel M.E., Sivakumar D., Sultanbawa Y. (2022). Hydrolysable Tannins, Physicochemical Properties, and Antioxidant Property of Wild-Harvested *Terminalia ferdinandiana* (Exell) Fruit at Different Maturity Stages. Front. Nutr..

[39] Uğur Y., Zengin R., Ernim C., Günhan Z.İ., Şalva E., Erdoğan S. (2024). Changes in the Phenolic, Melatonin, Sugar Contents and Antioxidant Capacity, Depending on Ripening Stage in Different Cornelian Cherry (*Cornus mas* L.) Fruits. Chem. Sel..

[40] Li Y., Li L., Zhang X., Mu O., Tian J., Yan J., Guo L., Wang Y., Song L., Yu X. (2023). Differences in total phenolics, antioxidant activity and metabolic characteristics in peach fruits at different stages of ripening. LWT-Food Sci. Technol..

[41] Çelik H., Özgen M., Serce S., Kaya C. (2008). Phytochemical accumulation and antioxidant capacity at four maturity stages of cranberry fruit. Sci. Hortic..

[42] Contreras C., Beaudry R. (2013). Lipoxygenase-associated apple volatiles and their relationship with aroma perception during ripening. Postharvest Biol. Technol..

[43] Tabaszewska M., Antoniewska A., Rutkowska J., Skoczylas Ł., Słupski J., Skoczeń-Słupska R. (2021). Bioactive Components, Volatile profile and in vitro antioxidative properties of *Taxus baccata* L. red arils. Molecules.

[44] Jetti R.R., Yang E., Kurnianta A., Finn C., Qian M.C. (2007). Quantification of selected aroma-active compounds in strawberries by headspace solid-phase microextraction gas chromatography and correlation with sensory descriptive analysis. J. Food Sci..

[45] Menager I., Jost M., Aubert C. (2004). Changes in physicochemical characteristics and volatile constituents of strawberry (Cv. *Cigaline*) during maturation. J. Agric. Food Chem..

[46] Oliveira I., Baptista P., Malheiro R., Casal S., Bento A., Pereira J.A. (2011). Influence of strawberry tree (*Arbutus unedo* L.) fruit ripening stage on chemical composition and antioxidant activity. Food Res. Int..

[47] El Arem A., Guidob F., Behijaa S.E., Manelc I., Nesrine Z. (2011). Chemical and aroma volatile compositions of date palm (*Phoenix dactylifera* L.) fruits at three maturation stages. Food Chem..

[48] Khan M.A., Ahmad R., Srivastava A.N. (2017). Effect of ethyl acetate aroma on viability of human breast cancer and normal kidney epithelial cells in vitro. Integr. Med. Res..

[49] Jordán M.J., Margaría C.A., Shaw P.E., Goodner K.L. (2003). Volatile components and aroma active compounds in aqueous essence and fresh pink guava fruit puree (*Psidium guajava* L.) by GC-MS and multidimensional GC/GC-O. J. Agric. Food Chem..

[50] Li Z., Howell K., Fang Z., Zhang P. (2019). Sesquiterpenes in grapes and wines: Occurrence, biosynthesis, functionality, and influence of winemaking processes. Compr. Rev. Food Sci. Food Saf..

[51] Hui L.M., Zhao G.D., Zhao J.J. (2015). Delta-Cadinene inhibits the growth of ovarian cancer cells via caspase-dependent apoptosis and cell cycle arrest. Int. J. Clin. Exp. Pathol..

[52] El Omari N., Mrabti H.N., Benali T., Ullah R., Alotaibi A., Abdullah A.D.I., Goh K.W., Bouyahya A. (2023). Expediting multiple biological properties of limonene and α-pinene: Main bioactive compounds of *Pistacia lentiscus* L., essential oils. Front. Biosci. (Landmark Ed.).

[53] Padilla-Jimenez S.M., Angoa-Pérez M.V., Mena-Violante H.G., Oyoque-Salcedo G., Renteria-Ortega M., Oregel-Zamudio E. (2019). Changes in the aroma of organic blackberries (*Rubus Fruticosus*) during ripeness. Anal. Chem. Lett..

[54] Caleb O.J., Fawole O.A., Mphahlele R.R., Opara U.L. (2015). Impact of preharvest and postharvest factors on changes in volatile compounds of pomegranate fruit and minimally processed arils—Review. Sci. Hortic..

[55] Rutkowska J., Baranowski D., Antoniewska-Krzeska A., Kostyra E. (2023). Comparison of storage-related volatile profiles and sensory properties of cookies containing xylitol or sucrose. Foods.

[56] Antoniewska A., Rutkowska J., Martinez Pineda M. (2019). Antioxidative, sensory and volatile profiles of cookies enriched with freeze-dried Japanese quince (*Chaenomeles japonica*) fruits. Food Chem..

[57] Du X., Song M., Baldwin E., Rouseff R. (2015). Identification of sulphur volatiles and GC-olfactometry aroma profiling in two fresh tomato cultivars. Food Chem..

[58] Re R., Pellegrini N., Proteggente A., Pannala A., Yang M., Rice-Evans C. (1999). Antioxidant activity applying an improved ABTS radical cation decolorization assay. Free Radic. Biol. Med..

[59] Van den Dool H., Kratz P.D. (1963). A generalization of the retention index system including linear temperature programmed gas–liquid partition chromatography. J. Chromatogr..

